# Perceived weight stigma in healthcare settings among adults living with obesity: A cross‐sectional investigation of the relationship with patient characteristics and person‐centred care

**DOI:** 10.1111/hex.13954

**Published:** 2024-01-15

**Authors:** Paige I. Crompvoets, Anna P. Nieboer, Elisabeth F. C. van Rossum, Jane M. Cramm

**Affiliations:** ^1^ Department of Socio‐Medical Sciences Erasmus School of Health Policy & Management, Erasmus University Rotterdam Rotterdam The Netherlands; ^2^ Department of Internal Medicine Division of Endocrinology, Erasmus MC, University Medical Center Rotterdam Rotterdam The Netherlands; ^3^ Obesity Center CGG Erasmus MC, University Medical Center Rotterdam Rotterdam The Netherlands

**Keywords:** obesity, person‐centred care, weight stigma

## Abstract

**Introduction:**

Patients living with obesity often experience weight stigma in healthcare settings, which has worrying consequences for their healthcare experiences. This cross‐sectional study aimed to: (1) provide an overview of stigmatising experiences in healthcare settings reported by adults living with varying classes of obesity, (2) identify associations among patient characteristics and perceived weight stigma and (3) investigate the association between perceived weight stigma and person‐centred care (PCC).

**Methods:**

Dutch adults living with obesity classes I (body mass index [BMI]: 30 to <35 kg/m^2^; *n* = 426), II (BMI: 35 to <40 kg/m^2^; *n* = 124) and III (BMI: ≥40 kg/m^2^; *n* = 40) completed measures of perceived weight stigma in healthcare settings and PCC. Descriptive, correlational and multivariate analyses were conducted.

**Results:**

Of patients living with classes I, II and III obesity, 41%, 59% and 80%, respectively reported experiences of weight stigma in healthcare settings. Younger age, greater obesity severity and the presence of chronic illnesse were associated with greater perceived weight stigma. Greater perceived weight stigma was associated with lower PCC.

**Conclusion:**

The results of this study emphasise the significant role of weight stigma in the healthcare experiences of patients living with obesity. Reducing weight stigma is expected to improve PCC and the overall quality of care for these patients. Minimising weight stigma will require efforts across various healthcare domains, including increasing awareness among healthcare professionals about sensitive communication in weight‐related discussions.

**Patient Contribution:**

Our sample consisted of patients living with obesity. Additionally, patients were involved in the pilot testing and refinement of the PCC instrument.

## 1 INTRODUCTION

While the global prevalence of obesity continues to increase, many patients living with this chronic condition are dissatisfied with their healthcare experiences and treatment outcomes.^1^, ^2^, ^3^ The focus of care for these patients is often limited to the achievement of weight loss, even though established guidelines advocate for a more comprehensive approach that addresses not only weight management but also the prevention of complications, management of comorbidities and improvement of overall well‐being and quality of life.^4^, ^5^ Despite the existence of such guidelines, many patients living with obesity receive inadequate medical attention and support, frequently feeling unheard and perceiving that healthcare professionals do not take their medical concerns seriously because of their weight.^6^ Consequently, many of these patients report weight‐related discrimination or bias, commonly referred to as weight stigma, during their interactions with healthcare professionals.^7^ Link and Phelan's^8^, ^9^ widely accepted theoretical framework holds that stigma arises when its components of labelling, stereotyping, separation, status loss and discrimination co‐occur in a power‐imbalanced situation that allows them to unfold. According to this theory, the labelling of individuals or groups as different, association of negative stereotypes with those labels and separation of these people into a distinct category (e.g., ‘them vs. us’) leads to status loss and discrimination. In healthcare settings, weight stigma can manifest through healthcare professionals' endorsement of negative stereotypes, exhibition of prejudicial attitudes and engagement in discriminatory behaviours toward patients because of their weight. Research indicates that this phenomenon extends across a wide range of healthcare professionals, including physicians, nurses, medical students, fitness professionals, dieticians and obesity specialists.^10^, ^11^, ^12^

Weight stigma has worrying consequences for the healthcare experiences of patients and may undermine the provision of person‐centred care (PCC), defined as ‘care that is respective of patients' preferences, needs and values and ensures that patient values guide all clinical decisions’.^13^ Patients who receive care in accordance with person‐centred principles tend to express greater satisfaction with care and achieve better treatment outcomes.^14^ However, the delivery of PCC to patients living with obesity may be hindered by healthcare professionals' negative attitudes and stereotyping of these patients because of their weight. A review highlighting the implications of weight stigma revealed that healthcare professionals holding such negative attitudes often exhibit reduced engagement in person‐centred communication, express less respect for patients living with obesity, allocate less time to their care and fail to provide adequate diagnostic testing and treatment options.^15^ The review further revealed that patients may, in return, experience elevated stress, withdraw from active participation in healthcare, and adhere poorly to professional recommendations due to mistrust. More recently, a multinational study documented several adverse healthcare experiences in response to weight stigma; patients reported increased judgement from physicians, reduced quality of healthcare encounters and diminished respect from physicians, along with less attention to their concerns.^16^ Experiencing weight stigma was also associated with attending fewer routine medical check‐ups and increased avoidance of healthcare services. Despite documentation of these adverse implications of weight stigma on patients' healthcare experiences, the link between patient experiences with weight stigma in healthcare settings and PCC is less well established.

Furthermore, there are gaps in the literature regarding the weight stigma experiences of patients living with obesity in healthcare settings. Existing evidence, primarily from the United States, highlights the prevalence of weight stigma, yet detailed data on the frequency and nature of these experiences are limited.^10^, ^16^ A few studies have shed light on the most common types of weight stigma experienced by patients. For example, adults in behavioural weight‐loss programs reported few overt stigmatising incidents; their experiences with stigma tended to be more subtle, such as when doctors brought up weight when the patients found it to be irrelevant.^17^ Similar findings have been reported for underserved women living with obesity seeking care in health centres.^18^ However, more research is needed to extend these findings to more diverse populations outside the United States, where weight stigma experiences may differ.

Additionally, the variability of weight stigma experiences based on patient characteristics remains unclear. Existing studies lack conclusive evidence on the link between sociodemographic factors and weight stigma, especially in the context of healthcare settings. While some findings suggest that females and younger individuals experience more weight stigma, these relationships lack consistency across studies.^17^, ^18^, ^19^, ^20^, ^21^, ^22^ Obesity is commonly classified by body mass index (BMI) into classes I (30 to <35 kg/m^2^), II (35 to <40 kg/m^2^) and III (≥40 kg/m^2^). Although weight stigma is often linked to higher obesity classes, evidence indicates that weight stigma in healthcare settings is experienced across obesity classes.^19^, ^20^ For instance, in a Swedish population‐based study, 25.8% of individuals living with class I obesity and 40.6% of those living with classes II and III obesity perceived weight stigma in healthcare settings.^23^ The impact of other health‐related factors remains largely unknown. Given that patients in poorer health are particularly vulnerable to the receipt of suboptimal care, they may be at greater risk of experiencing weight stigma.^24^ To identify those who are most vulnerable to the implications of weight stigma, a better understanding of the attributes of patients most likely to encounter such stigma in healthcare settings is needed.

Currently, the relationship between weight stigma and PCC is not well established, and data on the frequency and nature of weight‐stigmatising experiences in healthcare settings among patients living with obesity are lacking. Furthermore, data on patient factors that contribute to the perception of weight stigma in such settings are limited. We aimed to fill these gaps by pursuing three objectives: (1) to provide an overview of stigmatising experiences in healthcare settings reported by adults living with varying classes of obesity, (2) to explore associations between patient characteristics and perceived weight stigma and (3) to investigate the link between perceived weight stigma and PCC.

## 2 METHODS

### 2.1 Participants

Data for this study were collected through the Longitudinal Internet Studies for the Social Sciences panel, a probability‐based online panel comprising roughly 6500 individuals from about 4700 households, selected from the Dutch population register. Participants in the panel receive monetary compensation for completing monthly web‐based questionnaires. Annually, a longitudinal core study is conducted within the panel, capturing repeated health measures. Household and respondent demographics are updated monthly by one household member. Households lacking a computer or internet connection are provided with such to facilitate participation. Quest software is used for data collection. The panel abides by the European ‘General Data Protection Regulation’ and complies with all relevant ethical regulations. Our questionnaire was distributed among all panel members aged 18 years or older with a BMI of 30 kg/m^2^ or higher (*n* = 896) in July 2022, generating a total of 732 responses (82% response rate). A compensation of 4 euro was given to all respondents upon completion of the survey. BMI was calculated using self‐reported data on height and weight gathered during the latest wave of the annual health survey administered in November and December 2021. Outliers in BMI were identified by comparing the current wave's data with weight and height information from at least three previous waves, resulting in the exclusion of five cases with implausible weight values (e.g., 176 kg, compared to 76 kg in 2020 and preceding years). Furthermore, an examination of survey completion times led to the exclusion of seven respondents who completed the questionnaire faster than was deemed possible for accurate responses. Finally, data from 130 participants who responded ‘I do not know/not applicable’ to all PCC‐related items were excluded from the analysis, resulting in a final sample of 590 participants. Sample characteristics are provided in Table 1.

**Table 1:** Characteristics of the study sample (*n* = 590).

	Range	% Or mean (SD)
Sex (female)		57.1%
Age	18–92	59.22 (14.85)
Marital status (single)		34.2%
Education		
Low		33.2%
Intermediate		36.6%
High		30.2%
BMI	30–59	33.37 (3.88); 32 (4)^a^
30 to <35 kg/m^2^ (class I obesity)		72.2%
35 to <40 kg/m^2^ (class II obesity)		21.0%
≥40 kg/m^2^ (class III obesity)		6.8%
Chronic illness^b^		60.2%
SSHC^c^	0–48	3.03 (6.21); 0 (3)^a^
Person‐centred care^d^	1.8–5	3.83 (0.59)

Abbreviations: BMI, body mass index; IQR, interquartile range; SD, standard deviation; SSHC, Stigmatising Situations in Healthcare score.

^a^ Mean (SD); median (IQR).

^b^ Other than obesity.

^c^ Derived by summing all item scores (0 [never] to 3 [more than twice]), maximum = 48.

^d^ Derived by averaging dimension scores, range 1–5.

### 2.2 Measures

#### 2.2.1 Perceived weight stigma in healthcare settings

We assessed participants' perceptions of weight stigma in healthcare settings using a modified version of the 20‐item Stigmatising Situations in Healthcare (SSHC) questionnaire, which measures patients' experiences of weight stigma at a particular practice site.^18^ To broaden the instrument's applicability to a wider healthcare setting, we replaced specific terms like ‘doctor’ with ‘healthcare professional’ to make the questionnaire more inclusive. A strong overlap among some of the items allowed for the elimination of three items (‘Having nurses make negative remarks, ridicule you or call you names’, ‘Having medical staff make negative comments about weight to others’ and ‘Having office staff, for example, a front desk receptionist, make negative remarks to you’). We also excluded one item that was deemed unsuitable for the study's purpose (‘A doctor saying weight is a health problem when you are in good health’), as the World Health Organization and European Commission define obesity as a disease, even in the absence of complications.^25^ All adjustments were made in accordance with the expert opinion of an internist–endocrinologist and professor in the field of obesity and stress research who is involved in the provision of care to patients living with obesity and policy advice at the national and international level. The modified questionnaire had 16 items (Table 2). Participants were asked to rate each item on a 4‐point scale (never [0], once [1], twice [2] and more than twice [3]) to indicate how frequently the situation had occurred to them in a healthcare setting. An overall score was calculated by summing all item scores. Cronbach's *α* for the 16‐item measure was calculated at .91.

**Table 2:** SSHC items reported by patients living with obesity classes I, II and III.

How often has this happened to you?	At least once *n* (%)		
	Class I obesity (*n* = 426)	Class II obesity (*n* = 124)	Class III obesity (*n* = 40)
1. A healthcare professional blaming unrelated physical problems on your weight.	109 (25.6%)	49 (39.7%)	25 (62.5%)
2. A healthcare professional makes cruel remarks, ridicules you or calls you names.	26 (6.1%)	10 (8.2%)	10 (25%)
3. A healthcare professional recommending a diet even if you did not intend to discuss weight.	79 (18.5%)	41 (33.2%)	21 (52.5%)
4. Not being able to find medical equipment, such as blood pressure cuffs or gowns that fit you.	18 (4.1%)	8 (6.5%)	11 (27.5%)
5. A healthcare professional telling you to lose weight but not providing weight loss treatment options or advice on how to get help for weight loss.	76 (17.9%)	36 (29.2%)	19 (47.5%)
6. Being stared at by medical staff when you go to the doctor's office.	19 (4.4%)	9 (7.3%)	7 (17.5%)
7. Having healthcare professionals suggest diets to you without you asking for advice.	52 (12.3%)	23 (18.7%)	15 (37.5%)
8. Overhearing medical staff make rude comments about you.	18 (4.2%)	7 (5.9%)	9 (22.5%)
9. When you are weighed on a scale, the scale is not suitable for your weight.	9 (2.2%)	5 (4.3%)	7 (17.5%)
10. When you are weighed on a scale, the medical staff makes negative comments about your weight.	14 (4%)	9 (7.4%)	6 (15%)
11. Not being able to fit in chairs in the waiting room.	8 (1.9%)	7 (5.9%)	13 (32.5%)
12. A healthcare professional refusing to do an exam on you because of your weight.	4 (0.9%)	2 (1.9%)	7 (17.5%)
13. A healthcare professional assumes you overeat or binge‐eat because you are overweight.	50 (11.8%)	25 (19.9%)	17 (42.5%)
14. A healthcare professional assumes you have emotional problems because you are overweight.	26 (6.2%)	15 (11.7%)	14 (35%)
15. Being treated as less competent by healthcare providers because of your weight.	19 (4.5%)	8 (6.8%)	14 (35%)
16. Being treated as lazy by healthcare providers because of your weight.	21 (4.9%)	12 (9.4%)	14 (35%)
SSHC^a^ mean (SD); median (IQR)	2.0 (4.4), 0 (2)	3.8 (6.7); 1 (4)	10.6 (10.7), 8 (16)

Abbreviations: IQR, interquartile range; SD, standard deviation; SSHC, Stigmatising Situations in Healthcare score.

^a^ Derived by summing all item scores (0 [never] to 3 [more than twice]), maximum = 48.

#### 2.2.2 PCC for patients living with obesity

We measured PCC for patients living with obesity using a 40‐item instrument based on the eight dimensions of PCC: respect for patients' preferences, physical comfort, the coordination of care, emotional support, access to care, the continuity of care, the provision of information and education and the involvement of family members and friends.^26^, ^27^ The instrument builds on previous research that examined the importance of these dimensions to patients living with obesity,^28^ as well as research on PCC in other patient populations and healthcare settings.^29^, ^30^ The items were reviewed and discussed thoroughly, and adjustments were guided by relevant literature,^2^, ^5^, ^31^ consultation with two individuals living with obesity, and the expert advice of an internist–endocrinologist and professor specialised in obesity and stress research. Participants rated the items on a 5‐point Likert scale ranging from 1 (*totally disagree*) to 5 (*totally agree*), with the additional option to respond ‘I do not know/not applicable’. Average dimension scores were calculated when participants provided responses to ≥60% of the relevant items, and overall PCC scores were calculated for participants with at least five dimension scores by averaging those scores. The 40‐item model showed satisfactory‐to‐good fit, meeting structural equation modelling cut‐off criteria (comparative fit index = 0.90, standardised root mean square residual = 0.05, root mean square error of approximation = 0.06). The Cronbach's *α* values for the full instrument and subscales in this study were .92 and ≥.87, respectively.

#### 2.2.3 Patient characteristics

BMI values categorised participants into obesity classes: I (30 to <35 kg/m^2^), II (35 to <40 kg/m^2^) and III (≥40 kg/m^2^). Sociodemographic data included sex, age, marital status and education level. Marital status was classified as ‘single’ and ‘living with a partner’, with or without children. Education levels were ‘low’ (primary or lower vocational school), ‘intermediate’ (secondary or intermediate vocational school) and ‘high’ (higher vocational school or university). Additionally, to determine the presence of additional chronic illnesses, participants were asked to indicate (by ‘yes’ or ‘no’ response) whether they had any of 10 predefined conditions from a validated list (diabetes, cardiovascular diseases, heart failure, lung diseases, cancer, arthrosis, osteoporosis, chronic joint inflammation, depression and anxiety).^32^ They were also given the option to list any other chronic illnesses that they had.

### 2.3 Statistical analysis

Descriptive statistics encompassed frequency and percentage calculations for categorical variables, and mean with standard deviation for continuous variables. Given the positive skewness of BMI values and SSHC scores, median and interquartile range were also provided for these variables. Spearman coefficients were used to identify crude associations between SSHC scores and other study variables. To investigate multivariate relationships among patient characteristics and SSHC scores, a negative binomial regression model was applied due to the overdispersion detected in Poisson regression attempts. Exponential coefficients from the negative binomial model were used to calculate incidence rate ratios (IRR) along with their corresponding 95% confidence intervals. Finally, to investigate the relationship between SSHC and PCC scores while controlling for patient characteristics, multiple regression analysis was conducted. In case of multiple comparisons, Bonferroni corrections were applied to reduce the likelihood of type I error. An examination of missing values (items with a > 5% ‘not applicable’ response) revealed that participants without comorbid conditions had more missing data on some PCC items. In addition to standard complete‐case analysis (Tables SA1–SA3), multiple imputation was used to estimate overall associations among the variables. The Markov chain Monte Carlo algorithm was used to impute missing values 20 times (with 50 iterations), applying predictive mean matching as the imputation method. The analyses for this study were carried out using SPSS version 29.^33^

## 3 RESULTS

Descriptive statistics of all participant characteristics and study variables are depicted in Table 1.

### 3.1 Stigmatising experiences in healthcare

Table 2 shows the stigmatising experiences reported by individuals by obesity class. The percentages of participants reporting at least one weight stigma experience in a healthcare setting ranged from 41% for those living with class I obesity to 59% and 80% for those with classes II and III obesity, respectively. Across obesity classes, the most commonly reported experiences were healthcare professionals blaming unrelated physical problems on patients' weight (reported by 25.6%–62.5% of participants), recommending a diet even when patients did not intend to discuss weight (reported by 18.6%–52.5% of participants), and telling patients to lose weight but providing no weight‐loss treatment option or advice on how to get help for weight loss (reported by 17.9%–47.5% of participants).

### 3.2 Associations between patient characteristics and weight stigma

Older age correlated negatively with perceived weight stigma (*r* = –0.162, *p* < .001; Table 3). Higher BMIs (*r* = .266, *p* < .001) and having one or more chronic illnesses, excluding obesity, correlated positively with perceived weight stigma (*r* = .188, *p* < .001). These correlations remained significant after adjusting for other variables in the multivariate model. IRRs were used to interpret the effects of significant predictor variables. For age, the IRR of 0.98 revealed that for every 1‐year increase in age, the incidence rate of reporting weight stigma decreased by approximately 2%. Obesity class IRRs indicated that individuals living with class II obesity were about 1.88 times more likely to report weight stigma compared to class I, while those with class III obesity were about 4.57 times more likely compared to class I. Finally, individuals with one or more chronic illnesses, excluding obesity, were about 2.07 times more likely to report weight stigma compared to those without additional chronic illnesses. No significant associations were observed between perceived weight stigma and sex, marital status or education level.

**Table 3:** Correlation and regression coefficients between patient characteristics and perceived weight stigma (SSHC score) among patients living with obesity (*n* = 590).

	Spearman correlation		Negative binomial model		
	*r*	*p*‐Values	*B* (SE)	Incidence rate ratio (95% CI)	*p*‐Values
Sex (female)	.065	.116	0.17 (0.10)	1.186 (0.968, 1.455)	.100
Age	−.162	<.001	−0.02 (0.01)	0.980 (0.972, 0.988)	<.001
Marital status (single)	.028	.500	0.05 (0.11)	1.048 (0.852, 1.290)	.654
Education^a^	.026	.535			
Intermediate			0.01 (0.12)	1.007 (0.791, 1.281)	.956
High			−0.11 (0.13)	0.896 (0.691, 1.160)	.403
BMI^b^	.266	<.001			
35 to <40 kg/m^2^ (class II obesity)			0.63 (0.12)	1.878 (1.480, 2.380)	<.001
≥40 kg/m^2^ (class III obesity)			1.52 (0.19)	4.572 (3.180, 6.573)	<.001
Chronic illness^c^ (one or more)	.188	<.001	0.73 (0.11)	2.073 (1.672, 2.570)	<.001

Abbreviations: BMI, body mass index; CI, confidence interval; SSHC, Stigmatising Situations in Healthcare.

^a^ Reference group = low education.

^b^ Reference group = 30 to <35 kg/m^2^ (class I obesity).

^c^ Other than obesity.

### 3.3 PCC

Perceived weight stigma correlated negatively with PCC (*r* = −.308, *p* < .001; Table 4). Across obesity classes, greater perceived weight stigma was associated with lower PCC scores. These correlations remained significant after applying a Bonferroni adjustment (*α* = .013). The correlation coefficient was slightly stronger for obesity class III (*r* = –.400, *p* < .001) compared to class I (*r* = –.311, *p* < .001) and class II (*r* = –.289, *p* = .002). After controlling for patient characteristics in the multivariate model and applying a Bonferroni correction (*α* = .006), perceived weight stigma was the only significant predictor of PCC (*B* = −0.04, *p* < .001; Table 5).

**Table 4:** Spearman correlation coefficients between perceived weight stigma (SSHC score) and person‐centred care by obesity class (*n* = 590).

	Person‐centred care		
	*n*	*r*	*p*‐Values
SSHC	590	−.308	<.001
BMI			
30 to <35 (class I obesity)	426	−.311	<.001
35 to <40 (class II obesity)	124	−.289	.002
≥40 (class III obesity)	40	−.400	.012

Abbreviations: BMI, body mass index; SSHC, Stigmatising Situations in Healthcare.

## 4 DISCUSSION

This study investigated the frequency and nature of weight‐stigmatising experiences in healthcare settings reported by patients living with obesity. The percentage of patients who encountered weight stigma ranged from 41% for those living with class I obesity to 59% and 80% for classes II and III, respectively. Younger age, greater obesity severity and the presence of one or more chronic illnesses, excluding obesity, were associated with greater perceived weight stigma. Greater perceived weight stigma was associated with lower PCC, underscoring the significant role of weight stigma in the healthcare experiences of patients living with obesity.

**Table 5:** Relationship between perceived weight stigma (SSHC score) and person‐centred care, while controlling for patient characteristics, among patients living with obesity (*n* = 590).

	Person‐centred care		
	*B*	SE	*p*‐Values
Intercept	3.819	0.127	<.001
Sex (female)	0.045	0.046	.329
Age	0.001	0.002	.463
Marital status (single)	−0.059	0.050	.238
Education^a^			
Intermediate	−0.076	0.057	.187
High	0.012	0.060	.841
BMI^b^			
35 to <40 kg/m^2^ (class II obesity)	0.065	0.058	.264
≥40 kg/m^2^ (class III obesity)	0.231	0.102	.024
Chronic illness^c^ (one or more)	0.073	0.049	.141
SSHC	−0.040	0.004	<.001

Abbreviations: BMI, body mass index; SSHC, Stigmatising Situations in Healthcare.

^a^ Reference group = low education.

^b^ Reference group = 30 to<35 kg/m^2^ (class I obesity).

^c^ Other than obesity.

Consistent with previous studies,^17^, ^18^ the most commonly reported experiences in this study were related to how the subject of weight loss was approached, such as healthcare professionals' provision of unsolicited dieting advice or instruction that patients lose weight without the offering of treatment options. Despite notable differences in the reported frequency, these types of experiences were reported by patients across obesity classes. While discussion of weight may be important to improve patient outcomes, patients often perceive that such conversations are not tailored to their specific needs and that healthcare professionals may offer recommendations based on oversimplified assumptions about obesity.^34^, ^35^ For instance, simply advising weight loss to patients who have been struggling with weight management for a long time without providing any form of support may indicate to the patients that professionals do not appreciate the complexity of their situations, leading them to feel dissatisfied and misunderstood. On the other hand, healthcare professionals often feel unequipped to address weight issues with patients and may avoid the topic altogether or fail to provide appropriate support.^36^ For professionals seeking guidance in initiating conversations about weight, there are solutions like the ‘5As of obesity management’ approach, which begins by seeking permission from patients to discuss weight.^37^ The implementation of such an approach is supported by a recent study, revealing that among 1697 individuals living with overweight or obesity, the majority preferred that healthcare professionals ask permission to talk about weight.^38^

The most frequently reported experience in this study was healthcare professionals' attribution of physical problems, which patients perceived to be unrelated, to their weight. This experience is not uncommon among patients living with obesity. Research indicates that patients with higher weights may receive less consultation time from physicans.^39^ This may reflect a tendency to assess patients primarily based on their weight. The frequent reporting of healthcare professionals linking weight to unrelated problems may also indicate that patients have limited awareness about the connections between obesity and numerous medical conditions, including cardiovascular disease, type 2 diabetes, various types of cancer and many other health concerns.^40^ Clinical guidelines recommend that patients should be informed about their illness and educated about associated health risks, which may include discussions about weight as a modifiable factor.^41^ However, our findings suggest that patients may perceive such discussions as unwarranted and stigmatising. Thus, healthcare professionals must communicate in a supportive manner that enables patients to understand the potential links between their weight and health complaints. They may benefit from training that enhances their communication skills, particularly when discussing weight with patients living with obesity. A review highlighting effective strategies for minimising weight stigma in healthcare underscores the importance of systematically addressing this issue in healthcare education and practice.^42^ Recommended interventions include prioritising early and continuous education for healthcare students, with an emphasis on the complex and multifactorial aetiology of obesity, and the explicit integration of discussions about weight stigma and its consequences.

Notable differences in reported weight stigma were observed among the different obesity classes, with a clear trend of increased likelihood of perceived weight stigma as obesity severity increased from class I to class III. This aligns with previous findings that patients living with more severe obesity face greater weight stigma in healthcare.^17^, ^18^, ^19^, ^20^, ^23^ When examining the nature of reported experiences within obesity classes, a broader range of experiences is observed among patients living with class III obesity. This included more frequent reports of negative remarks or ridicule, being treated as less competent or lazy and facing issues related to healthcare environments, such as inadequately sized chairs or ill‐fitting equipment. Similar experiences have been extensively documented, with examples ranging from demeaning and embarrassing interactions to dismissal and inaccessible healthcare environments.^2^, ^15^, ^31^, ^43^ Given the limited sample of patients living with class III obesity, caution is necessary in interpreting the reported frequencies in this study. Nonetheless, the findings offer valuable insight into the various forms of weight stigma that these patients may encounter, and suggest that actions need to be taken across healthcare domains to improve these patients' care experiences.

Additionally, this study revealed a link between chronic illness and perceived weight stigma. An explanation could be that these patients spend more time in healthcare settings, increasing their exposure to stigmatising experiences. Another explanation may be that individuals with multiple medical conditions face more weight stigma due to the cumulative effects of having multiple stigmatised conditions. For instance, a study involving patients dealing with both obesity and chronic pain revealed that some patients felt shame following interactions with healthcare professionals who blamed them for both health issues.^44^ Importantly, the study's cross‐sectional design prevents the drawing of conclusions about the directionality of the observed association, which may also reflect the harmful effects of weight stigma on physical health.^45^ Finally, younger age was associated with greater weight stigma in this study, adding to prior evidence concerning this connection.

After adjusting for patient characteristics, greater perceived weight stigma in healthcare settings was associated with lower PCC. This finding aligns with existing evidence highlighting the harmful effects of weight stigma on the healthcare experiences and quality of care of patients living with obesity.^15^, ^16^ PCC has been established as a pillar of high‐quality care and may be particularly significant for patients living with obesity, given their complex and heterogenous support needs.^13^, ^46^ However, our findings suggest that weight stigma hinders the provision of PCC to this population, underscoring the urgency to combat weight stigma within healthcare settings.

This study has several limitations. First, due to the cross‐sectional study design, we were unable to establish the causality of the relationships observed. Second, given that weight stigma is believed to have a more significant impact on the care provided to patients living with severe obesity, the strength of the observed associations may have been affected by the limited number of patients living with class III obesity in the sample. Additional research with a larger population is needed to confirm our findings. Additionally, BMI was calculated using self‐reported height and weight, collected roughly 6 months earlier. Despite cross‐verifying outliers using data from previous waves, we cannot exclude the potential of misclassifications in obesity severity due to measurement errors or BMI changes during this period. Finally, a considerable number of participants reported having had no encounter with weight stigma in a healthcare setting. The SSHC items may not have captured certain distinct or context‐specific experiences of weight stigma, potentially leading to the underestimation of the frequency of weight‐stigmatising experiences. To capture the full range and depth of patients' experiences with weight stigma in healthcare settings, additional data collection methods such as qualitative interviewing may be required.

## 5 CONCLUSION

This cross‐sectional study outlines the prevalent and varied experiences of weight stigma in healthcare settings among patients living with obesity. The findings emphasise the significant role of weight stigma in shaping the healthcare experiences of these patients. Addressing weight stigma is expected to improve PCC and the overall quality of care for those dealing with obesity. Effectively minimising weight stigma will likely require comprehensive efforts across healthcare domains. Increasing awareness among healthcare professionals about the importance of sensitive and supportive communication in weight‐related discussions seems to be particularly important.

## AUTHOR CONTRIBUTIONS

**Paige I. Crompvoets**: Conceptualisation; writing—original draft; writing—review and editing; formal analysis; methodology. **Anna P. Nieboer**: Conceptualisation; writing—review and editing; supervision; formal analysis; methodology. **Elisabeth F. C. Rossum**: Conceptualisation; writing—review and editing; supervision; methodology. **Jane M. Cramm**: Conceptualisation; writing—review and editing; supervision; formal analysis; methodology.

## CONFLICT OF INTEREST STATEMENT

The authors declare no conflicts of interest.

## Supporting information

Supporting information.

## Data Availability

The data that support the findings of this study are available from the corresponding author upon reasonable request.
