# Supplementary material for: Perceived weight stigma in healthcare settings among adults living with obesity: A cross‐sectional investigation of the relationship with patient characteristics and person‐centred care
Source: Health Expect. 2024 Jan 15;27(1):e13954. doi: 10.1111/hex.13954 (PMC10790109; doi:10.1111/hex.13954)
Supplement: Supplementary file 1 — Supporting information. [file HEX-27-e13954-s001.docx]

Appendix A

Table A1.

*Correlation and regression coefficients between patient characteristics and perceived weight stigma (SSHC score) among patient living with obesity based on complete-case analysis (*n *= 571)*

|  | Spearman correlation | | Negative binomial model | | |
| --- | --- | --- | --- | --- | --- |
|  | *r* | *p* | B(SE) | Incidence rate ratio (95% CI) | *p* |
| Sex (female) | 0.061 | 0.143 | 0.18 (0.11) | 1.200 (0.977, 1476) | 0.083 |
| Age | -0.158 | < 0.001 | -0.02 (0.01) | 0.98 (0.972, 0.987) | < 0.001 |
| Marital status (single) | 0.036 | 0.384 | 0.04 (0.11) | 1.044 (0.847, 1.286) | 0.688 |
| Education^a^ | 0.017 | 0.681 |  |  |  |
| Intermediate |  |  | 0.01 (0.12) | 1.003 (0.787, 1.278) | 0.981 |
| High |  |  | -0.14 (0.13) | 0.872 (0.674, 1.127) | 0.295 |
| BMI^b^ | 0.271 | < 0.001 |  |  |  |
| 35 to <40 kg/m^2^ (class II obesity) |  |  | 0.65 (0.12) | 1.905 (1.499, 2.421) | < 0.001 |
| ≥40 kg/m^2^ (class III obesity) |  |  | 1.54 (0.19) | 4.645 (3.206, 6.729) | < 0.001 |
| Chronic illness*^c^* (one or more) | 0.196 | < 0.001 | 0.76 (0.11) | 2.144 (1.727, 2.660) | < 0.001 |

*Abbreviations: BMI, body mass index; SSHC, Stigmatizing Situations in Healthcare.*

*^a^Reference group = low education*

*^b^Reference group = 30 to <35 kg/m^2^ (class I obesity)*

*^c^Other than obesity.*

Table A2.

*Spearman correlation coefficients between perceived weight stigma (SSHC score) and PCC by obesity class based on complete-case analysis (*n *= 429*)

|  |  | PCC | | |
| --- | --- | --- | --- | --- |
|  |  | *n* | *r* | *p* |
| SSHC |  | 429 | -0.366 | < 0.001 |
|  | BMI |  |  |  |
|  | 30 to <35 (class I obesity) | 304 | -0.380 | < 0.001 |
|  | 35 to <40 (class II obesity) | 94 | -0.320 | < 0.001 |
|  | ≥40 (class III obesity) | 31 | -0.327 | 0.073 |

*Abbreviations: BMI, body mass index; SSHC, Stigmatizing Situations in Healthcare; PCC, person-centred care.*

Table A3.

*Relationship between perceived weight stigma (SSHC score) and PCC, while controlling for patient characteristics, among patient living with obesity based on complete-case analysis (*n *= 422)*

|  | PCC | | |
| --- | --- | --- | --- |
|  | *B* | *SE* | *p* |
| Intercept | 3.833 | 0.156 | < 0.001 |
| Sex (female) | 0.050 | 0.056 | 0.376 |
| Age | 0.001 | 0.002 | 0.713 |
| Marital status (single) | -0.028 | 0.058 | 0.625 |
| Education^a^ |  |  |  |
| Intermediate | -0.068 | 0.068 | 0.319 |
| High | -0.021 | 0.073 | 0.778 |
| BMI^b^ |  |  |  |
| 35 to <40 kg/m^2^ (class II obesity) | 0.057 | 0.069 | 0.407 |
| ≥40 kg/m^2^ (class III obesity) | 0.214 | 0.123 | 0.081 |
| Chronic illness*^c^* (one or more) | 0.056 | 0.060 | 0.354 |
| SSHC | -0.040 | 0.004 | < 0.001 |

*Abbreviations: BMI, body mass index; SSHC, Stigmatizing Situations in Healthcare; PCC, person-centred care.*

*^a^Reference group = low education*

*^b^Reference group = 30 to <35 kg/m^2^ (class I obesity)*

*^c^Other than obesity*
